# Mitochondrial Cytochrome c Oxidase and F_1_F_o_-ATPase Dysfunction in Peppers (*Capsicum annuum* L.) with Cytoplasmic Male Sterility and Its Association with *orf507* and *Ψatp6*-*2* Genes

**DOI:** 10.3390/ijms14011050

**Published:** 2013-01-07

**Authors:** Jiaojiao Ji, Wei Huang, Chuanchuan Yin, Zhenhui Gong

**Affiliations:** 1 College of Horticulture, Northwest A&F University, Yangling 712100, Shaanxi, China; E-Mails: jijiao963@163.com (J.J.); xnkankan@163.com (W.H.); yinchuanc35@163.com (C.Y.); 2 State Key Laboratory for Stress Biology of Arid Region Crop, Northwest A&F University, Yangling 712100, Shaanxi, China

**Keywords:** *Capsicum annuum* L., cytoplasmic male sterility, *orf507*, cytochrome c oxidase, *Ψatp6-2*, F_1_F_o_-ATPase

## Abstract

Cytoplasmic male sterility (CMS) in pepper (*Capsicum annuum* L.) has been associated with novel genes in the mitochondria, such as *orf507* and *Ψatp6-2*. Plant sterility has been proved to result from the rearrangement of the mitochondrial genome. Previous studies have demonstrated that *orf507* is co-transcribed with the *cox* II gene, and *Ψatp6-2* is truncated at the 3′ region of the *atp6-2* that is found in the maintainer line. Until this time, little has been known about the relationship between the novel gene and the function of its corresponding enzyme in mitochondria from the CMS pepper line. Moreover, the aberrant function of the mitochondrial enzymes is seldom reported in pepper. In this study, we observed that anther abortion occurred after the tetrad stage in the CMS line (HW203A), which was accompanied by premature programmed cell death (PCD) in the tapetum. The spatiotemporal expression patterns of *orf507* and *Ψatp6-2* were analyzed together with the corresponding enzyme activities to investigate the interactions of the genes and mitochondrial enzymes. The two genes were both highly expressed in the anther. The *orf507* was down-regulated in HW203A (CMS line), with nearly no expression in HW203B (the maintainer line). In contrast, the cytochrome c oxidase activity in HW203A showed the opposite trend, reaching its highest peak at the tetrad stage when compared with HW203B at the same stage. The *Ψatp6-2* in the CMS line was also down-regulated, but it was up-regulated in the maintainer line. The corresponding F_1_F_o_-ATPase activity in the CMS line was gradually decreased along with the development of the anther, which showed the same trend for *Ψatp6-2* gene expression. On the contrary, with up-regulated gene expression of *atp6-2* in the maintainer line, the F_1_F_o_-ATPase activity sharply decreased after the initial development stage, but gradually increased following the tetrad stage, which was contrary to what happened in the CMS line. Taken together, all these results may provide evidence for the involvement of aberrant mitochondrial cytochrome c oxidase and F_1_F_o_-ATPase in CMS pepper anther abortion. Moreover, the novel *orf507* and *Ψatp6-2* genes in the mitochondria may be involved in the dysfunction of the cytochrome c oxidase and F_1_F_o_-ATPase, respectively, which are responsible for the abortion of anthers in the CMS line.

## 1. Introduction

Cytoplasmic male sterility (CMS) is an important crop trait that is widely used in hybrid breeding to produce non-functional pollen [1]. This trait has proven to be related to the abnormal function of the plant’s mitochondria [2]. In many cases, this characteristic is a consequence of intermolecular rearrangements in the mitochondrial DNA, which produce many novel open reading frames (ORFs) that are co-transcribed with conventional mitochondrial genes [2]. Many ORFs that are associated with CMS have been identified, such as *orf239* in the common bean [3], *pcf* in petunia [4], *orf507* in pepper [5,6], *orfB* in *Brassica juncea* [7], *orfH522* in sunflower [8], *orfH79* [9] and *orfZ79* [10] in rice, to name a few. CMS phenotypes can also be induced by the differential expression of mitochondrial genes under the influence of various nuclear backgrounds [11] or due to the absence of RNA editing, as in *atp9* of wheat [12], and a substoichiometric copy number of a chimeric gene. For example, the restoration of fertility in common bean is related to the suppression of the copy number of a substoichiometric CMS-associated ORF by the action of a restorer gene [13]. In pepper, the substoichiometric difference is also present. Two mitochondrial genes associated with CMS in pepper, namely *orf507* and *Ψatp6-2*, have been shown to exist even in the maintainer lines with low copy number substoichiometry, as is also the case with CMS-associated genes from radish and common bean [5,13].

Normal anther development progress has many important steps. First, the microspore mother cell is differentiated from the reproductive cells and will then undergo meiosis to give rise to a tetrad, at which time the tetrad can release the microspores. The mononuclear microspore further undergoes two rounds of mitosis to form a three-celled pollen grain consisting of a larger vegetative cell and two sperm cells [14]. During this process, several anther tissues undergo programmed cell death (PCD) in a precisely coordinated and temporal progression [15,16]. Generally, the tapetum undergoes cellular degradation during the development of normal pollen grains. The tapetal cell can provide nutrients for the development of the pollen cell and meanwhile release lipid components for the formation of the pollen exine [17]. In many CMS plants, the premature PCD of the tapetum cell is also followed soon after by the death of the immature microspores. In some CMS plants, the callose envelopes persist until the microspore tetrad stage, so the single nuclear microspore cannot be released and thus cannot form a pollen grain [18]. However, in other CMS plants, individual microspore could be produced, whereas the development of single nuclear microspore was arrested because of the absence of energy provided by the mitochondria and nutrition from the tapetum cells [19].

Pollen formation is a highly energy-consuming process. Because pollen is non-photosynthetic tissue, the necessary energy is supplied by mitochondria, especially in the case of developing pollen grains [20]. The mitochondrion, which is the site of both the tricarboxylic acid cycle and the oxidative phosphorylation pathway, plays a crucial role in energy and carbon metabolism in eukaryotic cells through its role in the oxidative phosphorylation complex (OPC) [21]. The OPC include five complexes that are located in the inner membrane of the mitochondria, which are complex I (NADH dehydrogenase), complex II (succinate dehydrogenase), complex III (cytochrome bc1 oxidoreductase), complex IV (cytochrome c oxidase) and complex V (F_1_F_o_-ATP synthase) [22].

Cytochrome c oxidase (EC 1.9.3.1) plays a crucial role in aerobic life due to its specific capability to accept an electron from cytochrome c and then transfer it to dioxygen to form water while pumping protons across the membrane. The electrochemical potential difference of protons is used to drive ATP synthesis [23]. The enzyme complex is composed of several metal prosthetic sites and 13 protein subunits in mammals, of which three are synthesized in the mitochondria. COX II is firmly bound to subunit I and is the only subunit to possess a polar domain containing the binuclear Cu_A_ center. The Cu_A_ binuclear center is reduced by cytochrome c and then passes an electron on to cytochrome a, which in turn passes an electron on to the cytochrome a_3_-Cu_B_ binuclear center and, finally, to the dioxygen [24].

In plants, the majority of ATP is synthesized by F_1_F_o_-ATP synthase [25]. Furthermore, in addition to ATP synthesis, the mitochondrial F_1_F_o_-ATP synthase also engages in ATP hydrolysis, depending on the cell’s physiological pH [26,27]. In yeast, ATP hydrolysis activity is regulated by F_1_-ATPase inhibitor (INH1-IF) and stabilizer proteins [27–30]. These proteins can inhibit the ATP hydrolysis of F_1_F_o_-ATP synthase when it is not needed to ensure a sufficient supply of ATP for cell activities, and when the impaired membrane potential is rectified [27–30]. CMS is associated with oxidative phosphorylation complex (OPC) dysfunction, resulting in insufficient ATP to meet energy needs for pollen development [31].

For instance, the F_A_d subunit of mitochondrial F_1_F_o_-ATP synthase in *Arabidopsis thaliana*, which is encoded by the *MGP1* gene, is essential for anther development [25]. In sunflower, the *orf52*2, homology to *atp8*, encodes a subunit of F_1_F_o_-ATP synthase and induces male sterility in the plant [32]. Some novel ORFs are co-transcribed with the normal gene that encodes the expected protein in mitochondria and can also affect the normal protein’s function. For example, in rice Boro II cytoplasm, *orf79* is co-transcribed with the *B-atp6* gene and encodes a cytotoxic peptide, which leads to male sterility [33]. A similar phenomenon has also been found in the homologous Honglian rice gene, which is called *orfH79*. The protein that is encoded by *orfH79* in rice interferes with the construction of F_1_F_o_-ATPase, leading to a decrease in ATPase activity and the abortion of pollen grains [9]. In pepper, the CMS cytoplasm was first isolated from an Indian *C. annuum* accession (PI164835) [34]. The candidate genes *orf456* or *orf507* and*Ψatp6-2* have been identified in CMS pepper [5,6,35]. The *orf456* gene, which is co-transcribed with the *coxII* gene that encodes a subunit of cytochrome c oxidase in mitochondria, can induce male sterility in *Arabidopsis thaliana*, at least when it is over-expressed [35]. Gulyas [6] found that because of the deletion (cytosine, C), the original stop codon of *orf456* changed to asparagine (N), so the translated fragment was extended, ending with a new termination codon, giving a total length of 507 bp, named *orf507*. The *Ψatp6-2* is a 3′-truncated form of wild-type *atp6-2*, which encodes a subunit of F_1_F_o_-ATP synthase in normal cytoplasm [36]. Intact F_1_F_o_-ATP synthase contains 13 subunits, most of which are nuclear-encoded, except for ATP6, ATP8, ATP9, ATP4 of F_0_ and the ATPA of F_1_, which is encoded by mitochondrial genes [37].

The two novel genes of interest have been widely used as selection markers in pepper production, and *orf456* has been shown to induce male sterility in *Arabidopsis thaliana*, but the definite function of the genes and the relationship of the novel gene and its corresponding mitochondrial enzyme activity are not clear. Meanwhile, mitochondrial enzyme dysfunction in pepper has barely been explored to date, though the CMS in pepper is believed to be associated with this dysfunction [36].

In this study, we focused on investigating the relationship between novel mitochondrial *orf507*, *Ψatp6-2*gene and mitochondrial cytochrome c oxidase and F_1_F_o_-ATP synthase enzyme in the OPC system of a CMS line, respectively. The gene expression patterns and the mitochondrial enzyme activity were measured, following the ascertaining of the anther abortion characteristics in pepper CMS line.

## 2. Results and Discussion

### 2.1. Confirmation of Pollen Phenotype in the Sterile and Maintainer Lines

**Figure 1:**
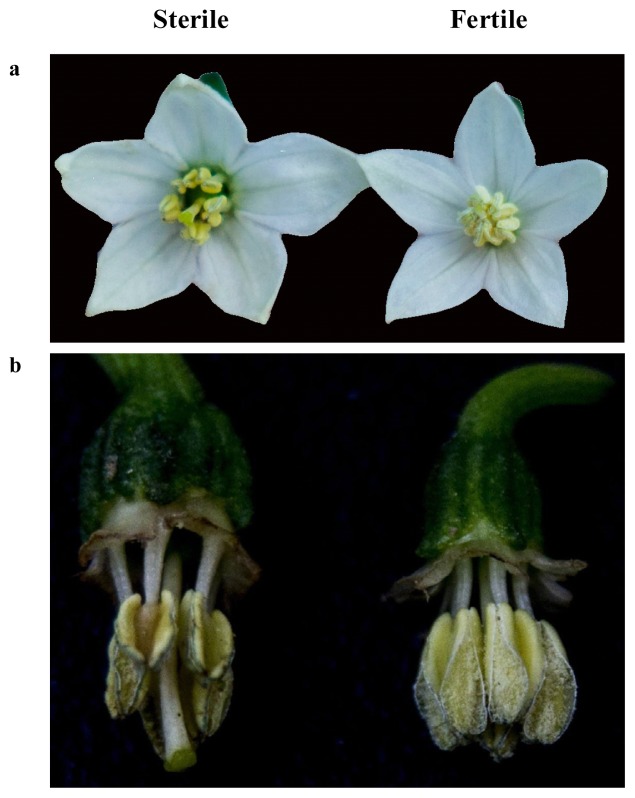
Morphological observation of flower in HW203A (CMS line) and HW203B (maintainer line). (**a**) Flowers from the sterile and fertile lines; (**b**) Anthers from the sterile and fertile lines.

The phenotypes of the flowers in both the sterile and maintainer lines were similar except for the number of pollen grains. The anthers in the sterile line hardly produced pollen grains, whereas the maintainer anthers were full of pollen grains (Figure 1).

### 2.2. Cytological Features of Anther Development in the Sterile and Fertile Pepper Lines

**Figure 2:**
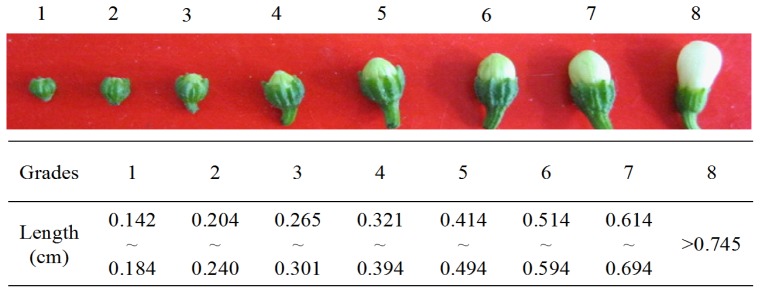
Grades dividing of the flower buds in pepper.

To study the relationship of premature PCD on the abortion of pollen in HW203A (sterile line), we compared the characteristics of pollen development with those from the HW203B (maintainer line). So far, studies of pepper anther development have been performed in many types of CMS system; moreover, the onset of abnormal development was reported to be varied from one system to another [18,38–40]. Shifriss and Frankel [38] and Horner and Rogers [18] observed that the sterile line could form an abnormal tetrad but could not produce normal pollen for the development of tapetal cells. Kaul [39] and Bhargawa [40] both thought that pollen development was abnormal at the beginning of meiosis. It is necessary to examine the pollen-abortive stage of our CMS line to further analyze gene expression and enzyme activity. Most prior reports focused on the initial cytological abnormalities during anther development in the sterile line to understand the potential causes of the CMS phenotype in pepper because the tetrads cannot then produce a mononuclear microspore. For our study, we first performed a paraffin section of the anther to observe its development. All the flower buds were divided into eight grades (Figure 2) according to the morphology of the buds [41], such as the length and the width of the pepper florets.

**Figure 3:**
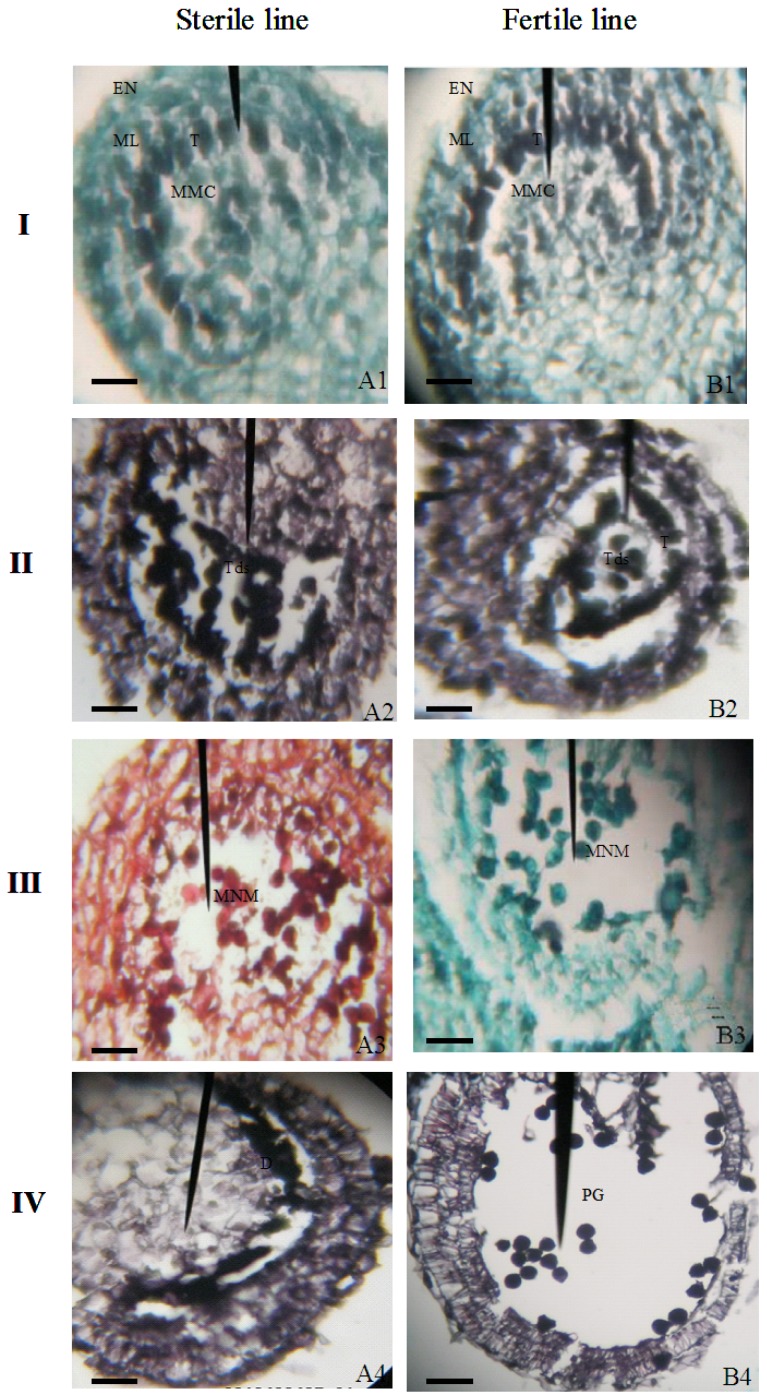
Anther development in florets of HW203A and HW203B. Four stages of anther development in the fertile line and the corresponding stages of development in the sterile line were compared using histological staining. The images show cross-sections through single locules: **I** Microspore mother cell stage; **II** Tetrad stage; **III** Mononuclear microspore stage; **IV** Mature pollen grain stage; **A**, Sterile line; **B**, Fertile line; T, tapetum; En, endothecium; ML, middle layer; MMC, microspore mother cell; Tds, tetrads; MNM, mononuclear microspore; D, degraded microspore; PG, pollen grain. Bars = 20 μm.

In histologically stained anther sections that were observed by using light microscopy, the abnormal development of CMS pepper became evident at the tetrad stage, when the tapetal cells started to degenerate. At the tetrad stage (grade 4 and 5), the microspores in the sterile line appeared normal relative to those of the maintainer line, though the tapeta began to disappear from the sterile line at this stage, whereas they persisted in the maintainer line (Figure 3II). When the florets of the CMS lines had reached a length that corresponded to grades 6 and 7, the tapetal cells thoroughly degenerated and the central nucleus microspores were released from the tetrad. The microspores were compressed in the center of the locule by the enlarged middle layer cells (Figure 3III(A3)). In the maintainer line, the tapeta also disappeared, and the microspores were uniformly distributed in the locule (Figure 3III(B3)). At grade 8, the middle layer cells further enlarged in the sterile line, and the sterile microspores were degraded and adhered to the residuum of the tapetal cells and were crushed into a lump of condensed material by the enlarged middle layer (Figure 3IV(A4)). Mature pollen grains were produced in the maintainer line at the same stage, and the middle layer cells were degraded (Figure 3IV(B4)).

**Figure 4:**
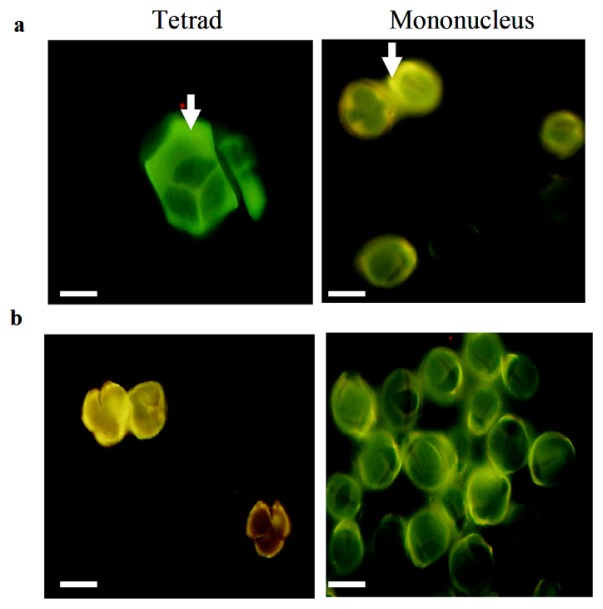
Aniline blue staining of the pollen at tetrad and mononucleus stages in HW203A (**a**) and HW203B (**b**). The white arrow indicates the callose. Bars = 20 μm.

To find if premature programmed cell death (PCD) caused the precocious callose degradation that was noted by Binh *et al.* [42], we stained the microspore with aniline blue. As shown in Figure 4, the stained microspores of HW203A and B had practically no differences between them, which implied that the premature PCD had nothing to do with the callose.

Together, these results show that the tetrad in HW203A could release single nucleus microspores, whereas no normal pollen grains were produced in the end, just as described by Wang *et al.* [43]. Although HW203A can produce single nucleus microspores, the tapetal cells were degraded early at the start of meiosis and had nearly completed its process by the tetrad stage. During normal pollen development, the anther tapetum starts to degrade at the tetrad stage and completely finishes when mature pollen forms [32]; the whole process is called programmed cell death (PCD). The programmed degradation of tapetal cells provides nutrients for pollen development such as proteins, lipids and others. Finally, the tapetal cell remnants and liposome are released to form a coat around the pollen surface. Therefore, we inferred that the premature PCD in HW203A might lead to a lack of nutrients and energy that is needed for normal pollen development, especially at the later stage, as is already known in sunflower [8]. The nutrient and energy deficiency of pollen might result in a precocious degradation, which would lead to the abortion of pollen at the mononuclear microspore stage in HW203A. During the development of the microgametophyte, the callose plays an important role in protecting the microspore and supporting the formation of the pollen exine wall [44]. Similar roles for the callose wall at the tetrad and mononuclear microspore stages in HW203A and HW203B indicated that the premature PCD did not affect the callose wall formation, and perhaps the abortion of pollen in the sterile line has a weak relationship to callose.

### 2.3. Gene Expression Analysis of *Cox II-orf507* and *Ψatp6-2*

Kim *et al.* [35] and Gulyas *et al.* [6] found that *orf507* and the *Ψatp6-2* [36] are associated with pollen formation in the pepper CMS line. Within the CMS line, *orf507*, which is a novel ORF at the 3′-end of the *cox* II gene, is co-transcribed with *cox* II, and *Ψatp6-2* is a pseudogene that is truncated at the *C*-terminal coding region when compared with the normal *atp6-2* from the maintainer line. Kim [35] showed that *orf456* was expressed in leaves and flowers, but it is unclear if the two genes could not or could only be slightly expressed in some certain tissues of the flower, and if they were temporally expressed. In our experiment, we analyzed the spatiotemporal expression patterns of *orf507* and*Ψatp6-2* in the sterile and maintainer lines to find if the two genes were related to the abortion of pollen grains by using reverse transcription PCR (RT-PCR) and real time PCR (qRT-PCR).

**Figure 5:**
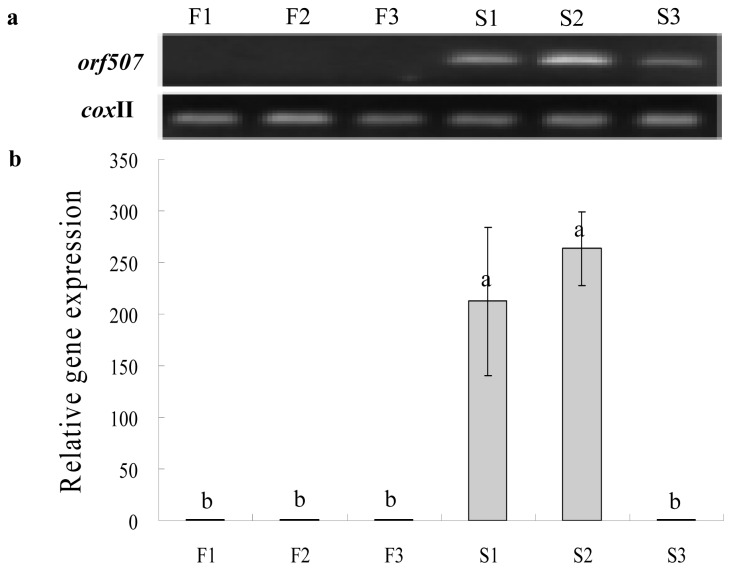
RT-PCR (**a**) and qRT-PCR (**b**) expression of *orf507* at different development stages of the pollen cells in sterile and fertile florets with *cox*II as the reference gene. Lanes F1~3 show florets at the microspore mother cell stage, tetrad stage and mononuclear microspore stage in the fertile line; S1~3 show florets at the corresponding stages in the sterile line. Data represent the means ± SD from three independent experiments. Statistically significant differences between the means were determined using Fisher’s LSD test (*p* < 0.05).

Gene expression analysis was then performed to investigate the differences in expression levels between CMS and the maintainer lines at different anther development stages, including the MMC (microspore mother cell), tetrad and mononuclear microspore stages. The cDNAs were reverse-transcribed from the total RNA of HW203A and HW203B florets at the three different development stages listed above. Pollen abortion started at the tetrad stage. The results showed that no or very faint expression products of *orf507* were detected in CMS flowers at the mononuclear microspore stage and that in the maintainer line, high-intensity expression was found at the tetrad and MMC stages (Figure 5). Gene expression levels in CMS line reached their highest peaks at the tetrad stage, which were significantly higher than at the mononuclear microspore stage; while they were a little higher than at the MMC stage, but with no significant difference. The high intensity expression before pollen abortion indicated that *orf507* was transcriptionally down-regulated and might be related to the induction of abortion.

**Figure 6:**
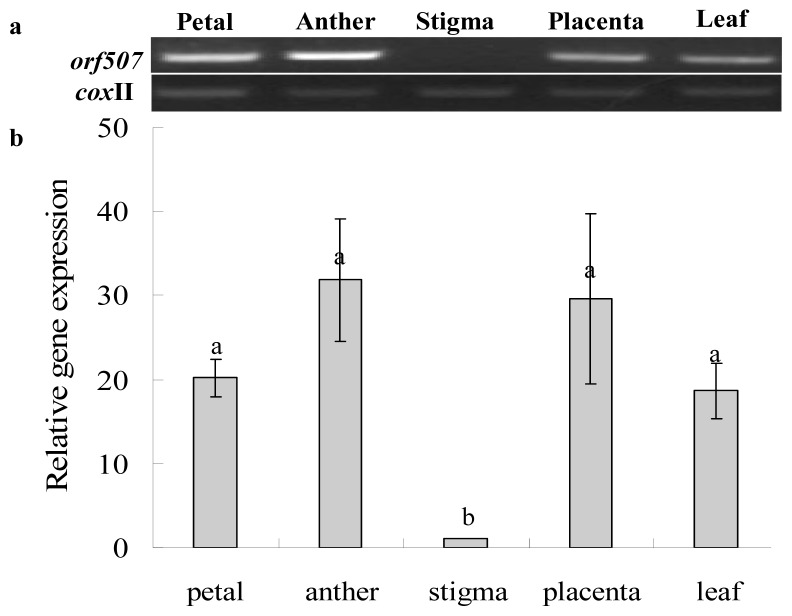
RT-PCR (**a**) and qRT-PCR (**b**) expression of *orf507* in different tissues from the pepper sterile line with *cox* II as the reference gene. Data represent the means ± SD from three independent experiments. Statistically significant differences between the means were determined using Fisher’s LSD test (*p* < 0.05).

To ascertain whether the gene was expressed in specific plant tissues, cDNAs from the petals, anthers, stigmas, placentas and leaves were used. The *orf507* transcripts were undetected or nearly so in the stigmas from the CMS line (Figure 6). The expression of *orf507* in the other four tissues had no significant difference, and they were highest in the anthers and placentas. This pattern indicated that *orf507* was not constitutively expressed in the flower and further demonstrated that the expression of this novel gene might cause injury to the anther or the microspore to some degree, with the highest expression in the anther.

**Figure 7:**
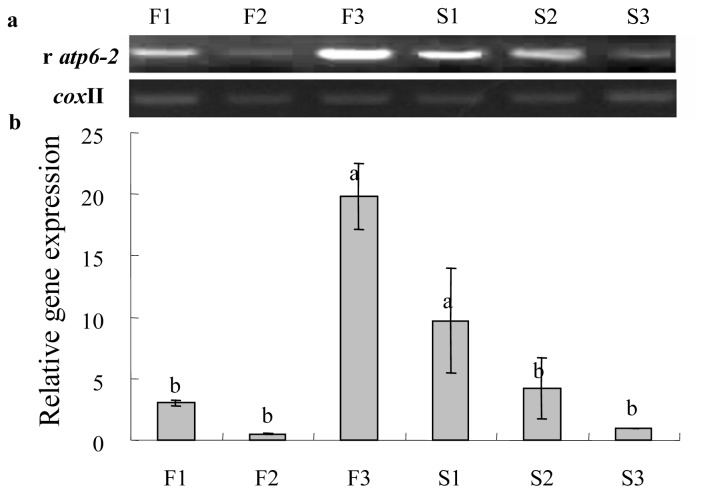
RT-PCR (**a**) and qRT-PCR (**b**) expression of the regular, non-truncated gene (r*atp6-2*) at different developmental stages of pollen cells in sterile and fertile florets with *cox* II as the reference gene. Lanes F1~3 indicate florets at the microspore mother cell stage, tetrad stage and mononuclear microspore stage in the fertile line; S1~3 show florets at the corresponding stage in the sterile line. Data represent the means ± SD from three independent experiments. Statistically significant differences between means were determined using Fisher’s LSD test (*p* < 0.05).

**Figure 8:**
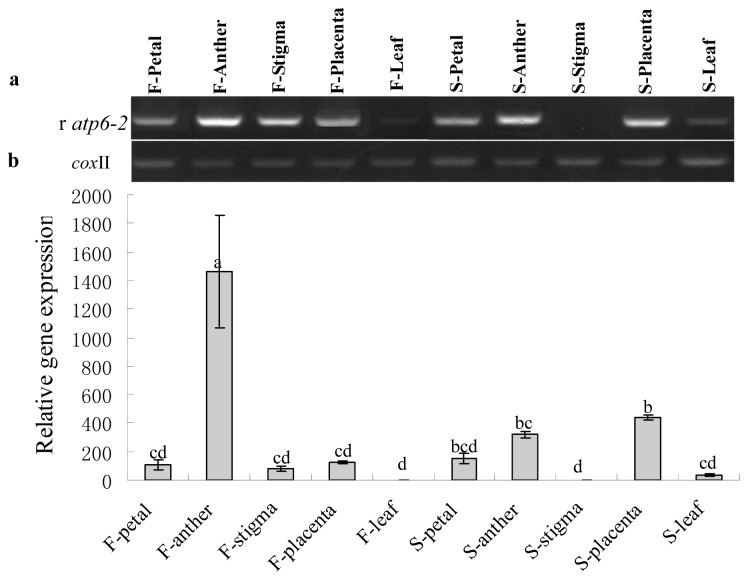
RT-PCR (**a**) and qRT-PCR (**b**) expression of r*atp6*-2 in different tissues of the sterile and fertile pepper lines with *cox* II as the reference gene, whereas F indicates the maintainer line and S is the CMS line. Data represent the means ± SD from three independent experiments. Statistically significant differences between the means were determined using Fisher’s LSD test (*p* < 0.05).

PCR primers were designed to amplify *ψatp6-2* in CMS plant. The primer set is expected to amplify *atp6-2* in maintainer plant. The expression of *atp6-2* in the maintainer plants was highest at the mononuclear microspore stage; however, it was highest at the MMC stage in the CMS line, and both were significantly higher than at other development stages. Meanwhile, the changing trend of gene expression in the CMS line was more or less contrary to what was found in the maintainer line. In concert with pollen development, the gene expression level was gradually decreased in the CMS, but it was dramatically increased in the fertile line after the tetrad stage (Figure 7). The opposing trends indicated that the *Ψatp6-2* in CMS was down-regulated, and conversely, the *atp6-2* was up-regulated in the maintainer line. Furthermore, spatial expression analysis showed that the pseudogene transcripts were not or were only weakly detected in the stigmas, petals and leaves both in the CMS and maintainer lines. However, the expression of *atp6-2* in anthers was significantly higher than in other tissues from the maintainer line. In contrast, the expression of *Ψatp6-2* in anther was relatively higher than other tissues in the CMS line and was slightly lower than in the placenta, as shown in Figure 8. We speculated that the *atp6-2* might have something to do with reproductive development, such as pollen development, and that *Ψatp6-2* might have a little opposite impact on pollen development when compared with *atp6-2* in normal cytoplasm.

In summation, both the CMS and maintainer lines showed that the two genes of interest were not, or were only weakly expressed in the stigma, which might imply that they did not affect the stigma during pollen development. Moreover, temporal expression study showed that the two genes were down-regulated in the CMS line. In the maintainer line, *orf507* was not expressed, whereas the opposite expression trend occurred for the *atp6-2* gene. Meanwhile, *orf507* and *Ψatp6-2* or *atp6-2* in normal cytoplasm were all highly expressed in the anther. Following these results, we speculated that *orf507* and*Ψatp6-2* might be mainly expressed in the tapetal cell or microspore cell. Their exact tissue specificity remains to be seen and could be further explored by RNA gel blot.

### 2.4. Evaluation of Cytochrome c Oxidase and ATPase Activity

CMS is closely connected with mitochondrial dysfunction in higher plants [45], especially with regards to energy metabolism. In rice, CMS-HL, *orfH79* transcripts have been shown to reduce ATPase stability and activity [9], but in rice, the CMS-ZA and *orfZ79* transcripts are associated with deficient NADH dehydrogenase activity but normal ATPase activity [10]. In a new *orf220*-type CMS line of *Brassica juncea* with down-regulated altered *atpA* gene, mitochondrial ATP synthesis decreased more than one-fold. [46]. However, few reports on mitochondrial enzyme activity in the CMS line of pepper have been published, although many novel mitochondrial genes that are caused by the rearrangement of DNA have been found and identified in this line. Given our current level of knowledge, we did not know if the novel genes were associated with a mitochondrial function for the corresponding enzyme. Kim *et al.* [35] transformed *orf456* into *Arabidopsis thaliana* by using tapetum-specific promoter *TA29*, and they observed a resulting defect in pollen-like grains in the exine wall. They speculated that the expression of this novel gene might cause premature programmed cell death in tapetal cells, which provided lipids and protein components for the formation of the pollen exine wall, especially callose. In addition to high *orf507* expression in buds before the tapetum degradation, we supposed that *orf507* had a bearing on premature PCD through an effect on the energy supply.

**Figure 9:**
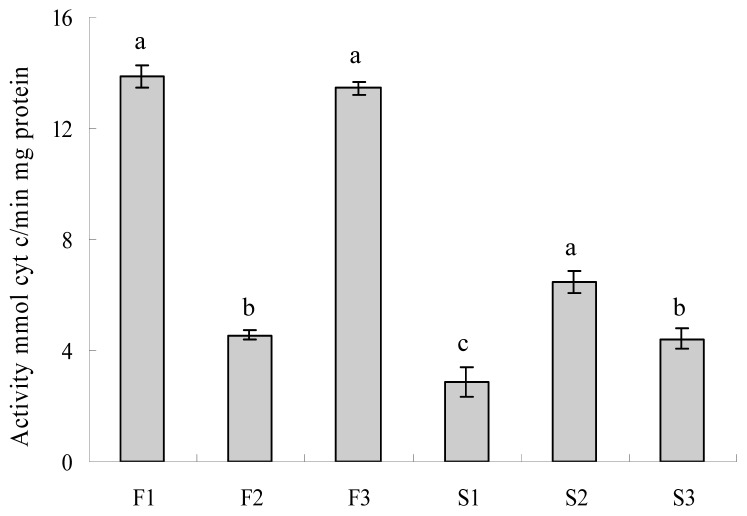
Activity of mitochondrial cytochrome c oxidase in pepper florets at different developmental stages. Lanes F1~3 show florets at the microspore mother cell, tetrad and mononuclear microspore stages in the fertile line; S1~3 denote florets at the corresponding stages from the sterile line. Data represent the means ± SD from three independent experiments. Statistically significant differences between means were determined using Fisher’s LSD test (*p* < 0.05).

To demonstrate the relevance of this gene to its relative enzyme activity, we analyzed cytochrome c oxidase and ATPase activity in the CMS and maintainer lines at different pollen development stages. Zhang *et al.* [9] demonstrated that *orfH79* contributes to a reduction in ATPase activity when co-transcribed with *atp6*. In the pepper CMS line, *orf507* is co-transcribed with *cox* II (which encodes a subunit of cytochrome c oxidase called COX II), just like the *orfH79* in rice. As shown in Figure 9, the enzyme activity of cytochrome c oxidase in the maintainer line is minimal at the tetrad stage, which is significantly lower than at the MMC and mononuclear microspore stages. An opposite trend was found in the CMS line. When the tapetum started to degenerate at the tetrad stage, the enzyme activity of cytochrome c oxidase reached the matrix. All these findings implied that the sterile line was cytochrome c oxidase-deficient. By comparative analysis of *orf507* gene expression level and cytochrome c oxidase activity, we speculated that the expression of *orf507* may affect the normal function of cytochrome c oxidase.

The function of COX II, with its Cu_A_ binuclear center, is to accept an electron from reduced cyt *c* and transfer it to cytochrome a in the respiratory chain [24]. It was hypothesized that ORF507 might interfere with the electron transmission from reduced cyt *c* to cyt *a*, or reduce the stability of the enzyme by affecting the interaction of COX II with other subunits of cytochrome c oxidase, such as the binding of COX II to COX I. Nevertheless, further evidence is still needed to define the relationship between this gene and cytochrome c oxidase. In the future, we can demonstrate the interactions between the *orf507* protein and cytochrome c oxidase using yeast two-hybrid assay.

In higher plants, F_1_F_o_-ATP synthase produces the bulk of the ATP. The two F_1_ and F_0_ parts are coupled in cells, and the intact F_1_F_o_-ATP synthase functions as ATPase and ATP synthase as regulated by the H^+^ concentration [47]. ATP6 is a subunit of F_1_, a water-soluble unit that contains the catalytic binding sites for ATP synthesis or hydrolysis [48,49]. This subunit plays an important role in the assembly of F_o_ with F_1_ because ATP6 has five transmembrane domains with an N terminus on the periplasmic side of the plasma membrane and a C terminus in the matrix compartment [49]. It was reported that mutations of *atp6*, such as the *atp6-orfH79* in Honglian rice and a single amino acid mutation in yeast mitochondria, could induce some interaction changes between ATP6 and other subunits of F_1_F_o_-ATP synthase; moreover, these interactions are needed to stabilize the assembly of F_o_ with F_1_ [50].

There are two copies of *atp6* in pepper, from which the sequence of *atp6-1* in the CMS line is the same as it is in the maintainer line. The other *atp6-2* has a different sequence in the CMS line, with a truncated 3′ region (*Ψatp6-2*) and an intact sequence in its counterpart.

**Figure 10:**
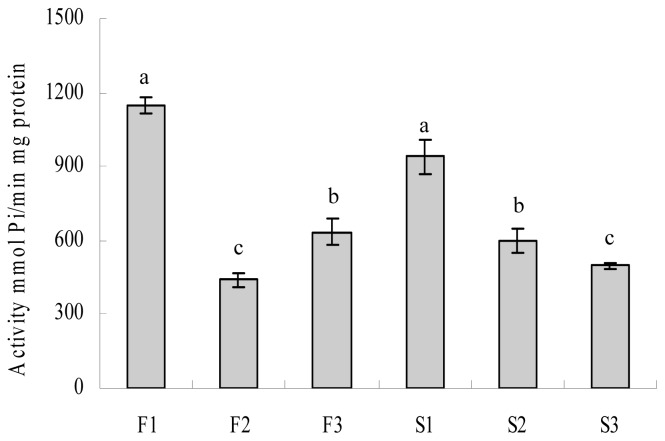
Activity of mitochondrial ATPase in pepper florets at different developmental stages. Lanes F1~3 show florets at the microspore mother cell, tetrad and mononuclear microspore stages in the fertile line; S1~3 indicate florets at the corresponding stages in the sterile line. Data represent the means ± SD from three independent experiments. Statistically significant differences between means were determined using Fisher’s LSD test (*p* < 0.05).

In a previous study, we showed that the transcription of *Ψatp6-2* was down-regulated in different pollen development stages, whereas the *atp6-2* was up-regulated in the maintainer line. With respect to the ATPase activity at the initial microspore mother cell stage, the ATPase activity in the maintainer line was the highest (F1) and was much higher than in the sterile line (S1). The enzyme activities in both the sterile and maintainer lines were significantly decreased after the MMC stage. After the tetrad stage, when the anther started to abort in the CMS line, the enzyme activity in the CMS line still gradually decreased, but it increased in the maintainer line. To analyze the trend as a whole, the up-regulated pattern of *atp6-2* and the gradually decreased ATPase activity might indicate that the normal gene might be involved in the inhibition of ATPase activity. The changing ATPase activity that was down-regulated in the CMS line was similar to the gene expression of *Ψatp6-2*, meaning that *Ψatp6-2* might increase the ATPase activity and promote ATP hydrolysis (Figure 10). These results showed that *atp6-2* and *Ψatp6-2* encoded different proteins with opposite effects on the function of ATPase. On the other hand, the changing ATPase activity happened after the beginning of the anther abortion of the CMS and maintainer lines, which may demonstrate that the mitochondrial F_1_F_o_-ATPase was aberrant in the CMS line, compared with in the maintainer line, and the dysfunction of the enzyme had something to do with the expression of *Ψatp6-2*.

The reverse ATP hydrolysis and synthesis of F_1_F_o_-ATP synthase is regulated by membrane electrochemical potential [25]. In yeast mitochondria F_1_F_o_-ATP synthase, the ATP6 of F_o_ forms the proton-conducting interface with the c9-15 ring. For functional F_1_F_o_-ATP synthase, ATP6 and subunits β and *α* consist of static subunits and the subunits γ, ɛ and c consist of rotating subunits. The reversible rotation of γ–ɛ–c subunits relative to the β and α catalytic subunits of F_1_ takes place during ATP synthesis (clockwise) and ATP hydrolysis (counterclockwise). The ATP6 and subunit c work as a stator to anchor the catalytic subunits of F_1_ to the membrane [51]. In view of our results, we supposed that ATP6-2 may be involved in switching ATP hydrolysis to ATP synthesis. The protein structure of F_1_F_o_-ATP synthase in the CMS line may have been changed by the expression of *Ψatp6-2*, and the changed structure may inhibit the coupled enzyme when switching to a clockwise direction to perform ATP synthesis, but still persists in hydrolyzing ATP. When compared with the increased ATPase hydrolysis activity in the maintainer line, the decreased ATPase activity following the tetrad stage may lead to insufficient ATP supplies for normal tetrad microspore or mononuclear microspore development, which ultimately results in the abortion of pollen in the CMS line. Nevertheless, more evidence is required to address this hypothesis. In view of the idea that ATP hydrolysis is not a simple reversible reaction of ATP synthesis from F_1_F_o_-ATP synthase, further demonstrations of ATP synthase activity between the CMS and maintainer lines will be performed in the future.

## 3. Experimental Section

### 3.1. Plant Materials and Growing Conditions

Pepper CMS line HW203A and its corresponding maintainer line HW203B, which are near-isogenic lines with similar nuclear genotypes and different cytoplasms, were provided by the Horticultural College of Northwest A&F University. Plants were grown in an unheated greenhouse during the summer, and the florets were harvested after flowering at different anther development stages.

### 3.2. Analysis of Pollen Development

The florets of fertile line HW203B and CMS line HW203A were divided into eight grades according to the length and width of the sepals and petals. The different floret grades were fixed, embedded, and sectioned using a procedure that was adapted from Peng *et al.* [41]. The paraffin section samples were observed under a light microscope. After the tetrad stage the microspore was stained with 0.02% aniline blue in 0.1 M PBS to observe the accumulation of callus around the microspore, which was examined with a fluorescence microscope under UV light.

### 3.3. Extraction of Mitochondria

The florets in the pollen mother cell, tetrad and single nuclear microspore stages were harvested, ground with a pestle in cold extraction buffer (0.4 M mannitol, 1 mM EDTA, 25 mM MOPS-KOH (pH 7.8), 10 mM KH_2_PO_4_, 0.1% BSA, 1% PVP 40 and 0.2% β-mercaptoethanol). The homogenate was filtered through a 200 mesh nylon strainer and then subjected to gradient centrifugation according to a procedure by Millar *et al.* [52]. The mitochondria pellets were resuspended in 1 mL of medium (0.4 M mannitol, 1 mM EDTA, 10 mM KH_2_PO_4_, 0.1% BSA). Protein yield was determined by UV-Vis spectroscopy.

### 3.4. Gene Expression Pattern Analysis

To analyze gene-specific expression, total RNA was isolated from the florets in the pollen mother cell, tetrad and single nuclear microspore stages from different tissues including the petals, anthers, stigmas, placentas and leaves using the Trizol method and was then purified with DNase I. The mRNA from each sample was subjected to reverse transcription using the PrimeScript™ RT-PCR Kit (Takara Biotech., Shiga, Japan) by combining the use of random hexamers and oligo dT primer. The primers that were used for RT-PCR and qRT-PCR were designed as indicated in Table 1, whereas *coxII* was used as the reference gene [6]. The primers that were used for gene expression analysis of *Ψatp6-2* and *atp6-2*, which is known here as r*atp6-2*, were designed according to the homologous sequences of the two genes. The qRT-PCR was performed using a SYBR Green Kit (Sigma, St. Louis, MO, USA). Reverse-transcription PCR (RT-PCR) was performed just like normal PCR. The PCR protocol was as follows: an initial step of 94 °C for 5 min, followed by 28 cycles of 94 °C for 30 s, 55 °C for 30 s, and 72 °C for 30 s, followed by a final elongation step of 72 °C for 10 min. The PCR products were detected by electrophoresis on a 1.5% agarose gel.

### 3.5. ATPase and Cytochrome c Oxidase Activity

The mitochondria pellets of florets at three different stages were lysed by freeze-thaw cycles with the addition of 0.05% Triton X-100 [32] and the lysate was used for the spectrophotometric analyses of enzyme activities. ATPase activity was estimated according to the method of Hans Bisswanger [53]. The activity of cytochrome c oxidase was analyzed as described by Prasad *et al.* [54], whereas the reaction mix contained 50 mM phosphate buffer, pH 7.0, 10 μM reduced cyt *c* (reduced with 10 mM sodium ascorbate) and 10 μL of protein in a final volume of 1.5 mL.

## 4. Conclusions

In conclusion, the abortion of the HW203A anther occurs after the tetrad stage, whereas the anther of the CMS line can produce a mononuclear microspore, but cannot form normal pollen grains. The expression of *orf507* and low enzyme activity in the CMS line has shown that *orf507* may be involved in the aberrance of the CMS cytochrome c oxidase, in addition to the opposing enzyme activity trend that was observed between the CMS and maintainer lines. Meanwhile, the down-regulated expression of *Ψatp6-2* and ATPase activity in the CMS line might indicate that the *Ψatp6-2* pseudogene produces a novel protein that could participate in the promotion of ATPase and cause persistent hydrolyzation of ATP. In the up-regulated *atp6-2* from the maintainer line, the ATPase was generally down-regulated, which suggests that ATP6-2 played a role in the inhibition of ATPase hydrolysis activity. The different changing trends of ATPase activity after anther abortion in both lines demonstrated that ATPase function was deficient in the CMS line. Moreover, ATPase dysfunction may cause the enzyme to have difficulty in hydrolyzing ATP to supply sufficient energy for the development of pollen after the tetrad stage. The dysfunction of the two mitochondrial enzymes will consequently result in an anther mitochondrial defect and upset the energy balance that is necessary for proper anther development. The mitochondrial enzyme dysfunction in the OPC system might cause the anther abortion in the CMS line. Therefore, we deduced that the novel *orf507* and *Ψatp6-2*gene might be associated with dysfunction of cytochrome c oxidase, F_1_F_o_-ATP synthase in this CMS type, respectively.

By identifying the gene expression of *orf507* and *Ψatp6-2* and their corresponding enzyme functions, it is hoped that this paper will help to reveal the full mechanism of the CMS phenomenon in chili pepper. On the basis of these studies, we will perform Virus-Induced Gene Silencing (VIGS) and genetic transformation to further explore the relationships of these two genes with mitochondrial enzymes in oxidative phosphorylation systems and CMS.

## Figures and Tables

**Table 1:** Oligonucleotides used for RT-PCR and qRT-PCR in this study.

Oligonucleotide	Sequence	Reference
*orf507* (forward)	5′-ATGCCCAAAAGTCCCATGTA-3′	[35]
*orf507* (reverse)	5′-AAAAGCGCTAAACAAATTGC-3′	This study
r *atp6-2* (forward)	5′-GCTTCGCCTTCGTATAGTAGTTC-3′	This study
r *atp6-2* (reverse)	5′-AGTCATAGTGCTCACCCTGTTTG-3′	This study
*coxII* (forward)	5′-ATGATTGTTCTAGAATGGCT-3′	[6]
*coxII* (reverse)	5′-TTATGGGATTAATTGATTGGATACCCG-3′	[6]
